# Effect of scan-time shortening on the ^11^C-PHNO binding potential to dopamine D_3_ receptor in humans and test–retest reliability

**DOI:** 10.1007/s12149-022-01819-4

**Published:** 2023-01-19

**Authors:** Keiko Matsunaga, Misato Tonomura, Kohji Abe, Eku Shimosegawa

**Affiliations:** 1 Department of Molecular Imaging in Medicine, Graduate School of Medicine, Osaka University, 2-1, Yamadaoka, Suita, Osaka, 565-0871 Japan; 2 Shionogi & Co., Ltd, 1-8, Doshomachi 3-Chome, Chuo-ku, Osaka, 541-0045 Japan

**Keywords:** PET, ^11^C-PHNO, Dopamine D_3_ receptor, Test–retest study, Scan-time optimization

## Abstract

**Objective:**

^11^C-PHNO is a PET radioligand most specific to dopamine D_3_ receptor (D_3_R). The long scan duration of 120 min used in quantification of ^11^C-PHNO binding to D_3_R in previous studies is challenging to subjects. The main objective of this study was to investigate the effects of shorter scan times on the binding of ^11^C-PHNO to D_3_R and test–retest reliability using the latest digital whole-body PET system.

**Methods:**

Two 120-min ^11^C-PHNO brain scans were performed in 7 healthy subjects using a digital whole-body PET/CT. The binding potential relative to non-displaceable tracer in the tissue (BP_ND_) of D_3_R-rich regions: the pallidum, ventral striatum (VST), substantia nigra (SN) and hypothalamus, were quantified using the simplified reference tissue model. The bias, correlation, and test–retest reliability of BP_ND_, which includes the test–retest variability (TRV) and intraclass correlation coefficient (ICC), were evaluated and compared between scans of shorter durations (40–110 min post-injection) and the original 120-min scan acquisitions.

**Results:**

Progressively, shorter scan durations were associated with underestimation of BP_ND_, slightly decreased correlation with 120-min derived BP_ND_, and decrease in test–retest reliability. The BP_ND_ values of the pallidum, VST and SN from the shortened 90-min scans showed excellent correlation with those derived from the 120-min scans (determination coefficients > 0.98), and the bias within 5%. The test–retest reliability of BP_ND_ in these regions derived from 90-min scan (TRV of 3% in the VST and pallidum, 7% in the SN and the ICC exceeded 0.88) was comparable to those obtained in previous 120-min studies using brain-dedicated PET scanners. In the hypothalamus, the BP_ND_ values obtained from scan-time less than 110 min showed bias larger than 5% and the TRV more than 9%.

**Conclusion:**

The scan-time shortening causes bias and decreasing test–retest reliability of ^11^C-PHNO BP_ND_. However, in the whole-body PET system, 90-min scan duration was sufficient for estimating the ^11^C-PHNO BP_ND_ in the D_3_R-rich striatum and SN with small bias and at the test–retest reliability comparable to those derived from 120-min scans using the brain-dedicated PET systems.

**Supplementary Information:**

The online version contains supplementary material available at 10.1007/s12149-022-01819-4.

## Introduction

The dopamine D_3_ receptor (D_3_R) was cloned about 30 years ago [1]. In the central nervous system (CNS), D_3_R is expressed in regions such as the pallidum, ventral striatum (VST), substantia nigra (SN) and hypothalamus [2–8]. The D_3_R is generally known to be involved in the regulation of cognitive, social, emotional, motivational and locomotor processes [9–11]. Use of D_3_R-selective agents is considered as a potentially effective treatment option for CNS diseases such as schizophrenia, drug abuse, Parkinson’s disease, and depression [11]. Therefore, D_3_Rs have become a promising target of drug research.

^11^C-( +)-4-propyl-9-hydroxynaphthoxazine (hereafter, ^11^C-PHNO) is a PET radioligand with affinity for dopamine D_2_ receptor (D_2_R) and D_3_R [12], but a 25- to 48-fold higher affinity for D_3_R than for D_2_R in vivo [13]. ^11^C-PHNO binding in D_2_R- and D_3_R-expressing regions depends on the proportional densities of the two receptors. D_3_R-specific binding ranges from 0% in the putamen and caudate, 20% in the VST, 50% in the thalamus, 60% in the pallidum, to 100% in the SN and hypothalamus in humans in vivo [14]. The amygdala is also known to express D_2/3_Rs with D_3_R fractions of 20–90% from the autoradiographic study of the human postmortem brain [7]. Until date, ^11^C-PHNO is the most specific PET radioligand available to assess D_3_R expression in humans [15]. ^11^C-PHNO binding in D_3_R-rich regions, such as the SN, is suitable for the evaluation of drugs acting on the D_3_R [16].

Theoretically, the longer the scan time, the more accurate the binding parameter estimations. Furthermore, shortening of the data acquisition time is also desirable from the point of view of comfort of the subjects. Ginovart et al. compared the striatal binding potential relative to non-displaceable tracer in the tissue (BP_ND_) of ^11^C-PHNO derived from 90-min scans and scans of shorter durations. While they reported that 80-min scans are sufficient for evaluation of the striatum, they did not investigate D_3_R-rich extrastriatal regions, such as the SN [17]. Another study reported the test–retest reliability of BP_ND_ in striatal and extrastriatal D_3_R-rich regions derived from 120-min scans [18]. Although supplemental data showed that the values of BP_ND_ were slightly underestimated in the 90-min scans as compared with 120-min scans, and the test–retest variability (TRV) in D_3_R-rich regions was slightly better in the 120-min scans, the effect on the intraclass correlation coefficient (ICC) has not yet been reported. Thus, the effects of shortened ^11^C-PHNO PET scan times on the binding parameters and test–retest reliability have not been fully investigated until date.

The previous human brain ^11^C-PHNO PET study with arterial blood sampling revealed that the two-tissue compartment model gave the stable estimation of BP_ND_, when K_1_/k_2_ fixed to the value obtained in cerebellum [17]. The BP_ND_ value derived from the simplified reference tissue model (SRTM), with the cerebellum as the reference region to estimate nonspecific binding, had an excellent correlation with those obtained from the two-tissue compartmental modeling. As a result, the SRTM has been used routinely as a noninvasive method in evaluating BP_ND_ of ^11^C-PHNO in clinical studies. Semi-quantitative analysis using the standardized uptake value ratio (SUVR) has been applied to the estimation of BP_ND_ in the receptor study [19]. The reduction of scan time via SUVR analysis is beneficial to subjects. However, the SUVR approach has not been reported in the estimation of ^11^C-PHNO BP_ND_ of brain dopamine receptors.

D_3_R-rich regions of the brain, such as the SN and hypothalamus, are small in volume and therefore susceptible to the partial volume effect and noise. PET scanners with a high spatial resolution are required for accurate assessment of the BP_ND_ in these regions. While brain-dedicated PET systems with a high spatial resolution were used in previous test–retest studies [18, 20, 21], no test–retest studies of ^11^C-PHNO performed using clinical whole-body PET systems have been reported. In addition, adverse effects associated with the administered mass dose are known in ^11^C-PHNO PET [22]. The injectable dose is limited by the mass of PHNO and the specific radioactivity, and the limited radioactivity could affect the image quality. Thus, it is clinically important to estimate the accuracy of measurement of the ^11^C-PHNO BP_ND_ using shorter scan protocols and a whole-body PET scanner.

In the present study, we assessed the effect of shorter scan times on the ^11^C-PHNO binding parameters and test–retest reliability in healthy human volunteers. In addition, we examined the applicability of SUVR analysis to BP_ND_ estimation. We also examined the feasibility of measuring ^11^C-PHNO binding to D_3_R at doses that rarely cause serious adverse effects, using a state-of-the-art digital whole-body PET/CT system.

## Methods

### Subjects

Eight healthy volunteers (6 men, 2 women; mean age: 37 $\pm$ 9 years, range: 22–49 years) were included in this study. Written informed consent was obtained from all the participants prior to the examinations. The absence of recent substance use was confirmed by urinary toxicology (SIGNIFY™ ER Drug Screen Test) on the days of screening, the 1^st^ and 2^nd^ PET scanning. The protocol of the present study was approved by the local Ethics Committee of Osaka University Hospital. Each subject underwent 2 PET scans separated by 12 $\pm$ 5 (6–22) days. In one of the 8 subjects, retest scan was aborted due to failure of the PET/CT scanner.

### Radiochemistry

^11^C-PHNO was prepared as previously reported [12]. The radiochemical purity was greater than 97.3% and the specific activity at the end of the synthesis was 70 $\pm$ 7 MBq/nmol.

### PET imaging

The PET examinations were conducted using a Digital Biograph Vision PET/CT system (Siemens Healthineers). This scanner has 8 rings composed of 38 detector blocks, each block containing 4 $\times$2 mini blocks; each mini block consists of a 5$\times$ 5 lutetium oxyorthosilicate array of 3.2 $\times$ 3.2 $\times$ 20 mm crystals coupled to a silicon photomultiplier array of 16 $\times$ 16 mm, yielding an axial field of view of 26.1 cm. The intrinsic spatial resolution is 3.6 mm in the transverse and 3.5 mm in the axial direction, in full-width at half maximum (FWHM) at a 1-cm offset from the center of the field of view. The National Electrical Manufactures Association (NEMA) sensitivity is 16.4 kcps/MBq, and a NEMA peak noise-equivalent count-rate is 306 kcps at 32 kBq/mL [23].

After low-dose CT scanning for attenuation correction, a bolus intravenous injection of ^11^C-PHNO (137 $\pm$ 14 MBq) was administered via a line placed in an antecubital vein, and the line was flushed with 10 mL of saline immediately after the tracer injection. The mean specific radioactivity of ^11^C-PHNO at the time of injection was 27 $\pm$ 3 MBq/nmol, and the mean injected mass was 20 $\pm$ 2 ng/kg (maximum, 24 ng/kg). The injected dose and mass did not differ significantly between the test and retest scans (paired t-test, P = 0.16 and 0.15, respectively) (Table 1).

**Table 1:** Synthesis and injection parameters (*n* = 8 subjects)

Parameter	Test scan	Retest scan	Variation^*^
Specific activity at the end of synthesis (MBq/nmol)	$68 \pm 6$	$72 \pm 8$	$7\mathrm{\% }\pm 15\mathrm{\%}$
Specific activity at the end of injection (MBq/nmol)	$26 \pm 2$	$27 \pm 3$	$4\mathrm{\% }\pm 13\mathrm{\%}$
Injected dose (MBq)	$132 \pm 5$	$142 \pm 18$	$7\mathrm{\% }\pm 13\mathrm{\%}$
Injected mass (ng/kg)	$20 \pm 2$	$21 \pm 2$	$4\mathrm{\% }\pm 5\mathrm{\%}$

*Computed as retest value/test value–1

List mode PET data were binned into the following frames 6 $\times$ 30 s; 3 $\times$ 1 min; 2 $\times$ 2 min; 22 $\times$ 5 min, with a total duration of 120 min following ^11^C-PHNO injection. Dynamic list mode data were reconstructed with an ordinary Poisson ordered-subset expectation maximization 3-dimensional iterative algorithm using 8 iterations, 5 subsets, with application of all corrections (attenuation, normalization, scatter, randoms, deadtime and time-of-flight), and a 2-mm FWHM Gaussian filter. Point spread function correction was not applied. The number of axial slices was 132. The resulting image size was 440$\times$ 440 $\times$ 132, with a voxel size of 0.825 $\times$ 0.825 $\times$ 2 mm.

### MR imaging

MR images of the brain were acquired on the same day as the test or retest PET scan to define the regions of interest (ROIs). MR imaging was performed in an Achieva 3.0-T system (Philips) with a circularly polarized head coil. MR images were acquired in an axial 3D spoiled gradient echo (SPGR) sequence, at 2.49 ms echo time, 6000 ms repetition time, and 15-degree flip angle. The image dimensions were 512 $\times$ 512 $\times$ 210 and pixel size was 0.47 $\times$ 0.47 $\times$ 1.0 mm.

### Quantification of the PET data

Regional time–radioactivity curve (TAC) computation was performed as reported in a previous study [18]. Individual MRIs were non-rigidly registered to the Montreal Neurological Institute (MNI) template [24] using the BioimageSuite software (version 1.3; http://www.bioimagesuite.org) [25]. Motion correction of dynamic PET images was performed by applying rigid registration of each frame image to an early summed image (0–10 min post-injection) using a 6-parameter mutual information algorithm (FLIRT, FSL 6.0, Analysis Group, FMRIB, Oxford, UK) [26, 27]. The early summed PET images were rigidly registered to individual MR images using a similar approach. We analyzed motion-corrected PET images in an MNI template space by applying two transformations: rigid transformation from individual PET images to individual MR images and nonrigid transformation from individual MR images to the MNI template.

The gray matter ROIs were taken from the Anatomical Automatic Labeling (AAL) template [28] delineated on a MR template [24]. Six ROIs were selected: the cerebellum (194 cm^3^ in template space), caudate (13 cm^3^), putamen (16 cm^3^), pallidum (4.6 cm^3^), amygdala (3.7 cm^3^), and thalamus (17 cm^3^). Extra ROIs corresponding to the hypothalamus (0.9 cm^3^) and VST (2.6 cm^3^) were also drawn on the template MRI. The ROIs in the hypothalamus and VST were drawn by reference to previous studies [14, 29], respectively. Finally, a SN template ROI (2.0 cm^3^) was also created in accordance with the methods described previously [18]. Regional TACs were obtained by applying the ROIs to transformed PET dynamic images on the template space. On the basis of a previous report [18], SRTM [30] was used to estimate the ^11^C-PHNO BP_ND,_ using the cerebellum as the reference region. BP_ND_ in each region was estimated using weighted least squares, with weights based on the noise-equivalent counts in each frame, using the PMOD software (version 3.8; PMOD Technologies; Zürich, Switzerland).

### TRV and ICC estimation

The TRV of BP_ND_ (noted as $\Delta$) was defined as follows:

1 $$\Delta {\mathrm{BP}}_{\mathrm{ND}} = 2\frac{{\mathrm{BP}}_{\mathrm{ND}}^{\mathrm{retest}} -{\mathrm{ BP}}_{\mathrm{ND}}^{\mathrm{test}}}{{\mathrm{BP}}_{\mathrm{ND}}^{\mathrm{retest}} + {\mathrm{BP}}_{\mathrm{ND}}^{\mathrm{test}}},$$

where ${\mathrm{BP}}_{\mathrm{ND}}^{\mathrm{test}}$ and ${\mathrm{BP}}_{\mathrm{ND}}^{\mathrm{retest}}$ are the BP_ND_ values obtained from the test and retest scans, respectively. The mean $\Delta$ BP_ND_ across subjects (denoted as m($\Delta$ BP_ND_)), the standard deviation (SD) of the $\Delta$ BP_ND_ across subjects (denoted as $\upsigma$($\Delta$ BP_ND_), and the mean across subjects of the absolute value of the $\Delta$ BP_ND_ (denoted as m(|$\Delta$ BP_ND_|) were computed. The m($\Delta$ BP_ND_) denotes the trend between the test and retest scans. $\upsigma$($\Delta$ BP_ND_) and m(|$\Delta$ BP_ND_|) are indices of variability [18]. The ICC was also computed by reference to a previous study [31].

### Effect of scan duration

For evaluating the effects of scan shortening, we created data from 40-min, 50-min, 60-min, 70-min, 80-min, 90-min, 100-min, and 110-min scans by deleting frames from the 120-min scans. Then, we evaluated the effects of scan shortening on the BP_ND_, TRV, and ICC.

First, the correlations between the BP_ND_ values obtained from the 120-min scan and scans of shorter durations were investigated. Linear regression fitting and coefficients of determination (r^2^) were used to compare the BP_ND_ values obtained from the shortened scans and those obtained from the original 120-min scans. Then, we investigated the BP_ND_ bias from scans of shorter durations in the same population. The bias to assess overestimation or underestimation due to scan-time shortening was calculated using the following equation:

2 $$\mathrm{ Bias }=\frac{{\mathrm{BP}}_{\mathrm{ND\, derived\, from\, shorter\, scan}}- {\mathrm{BP}}_{\mathrm{ND\, derived\, from }\,120\,\mathrm{minscan}}}{{\mathrm{BP}}_{\mathrm{ND\, derived\, from }\,120\,\mathrm{minscan}}}.$$

Finally, from the data of 7 subjects in whom the set of 120 min test and retest scans could be completed, we determined the TRV and ICC for each scan duration. From the data of the 7 subjects in whom test–retest scans were completed and 1 subject in whom the test scan was completed but the retest scan was aborted, we assessed the BP_ND_ bias and analyzed the correlations of the BP_ND_ values obtained between the 120-min scans and scans of shorter durations (*n* = 8 subjects, 15 scans in total). We defined the minimal scan duration for reliable estimation of BP_ND_ as follows: r^2^ > 0.9, bias within 5% [32], ICC > 0.7, and TRV over the values of the previous study derived from 120-min scan [18].

### SUVR analysis

We calculated the SUVR using 30-min images from 90 to 120 min post-injection, as follows:

3 $$\mathrm{SUVR }=\left({\int }_{90min}^{120min}C\left(t\right)dt\right)/\left({\int }_{90min}^{120min}{C}_{r}\left(t\right)dt\right),$$

where *C* and *C*_*r*_ are the radioactivity concentration of the target region and cerebellum, respectively. We evaluated the correlations between SUVR – 1 and BP_ND_.

### Statistical analysis

We used the statistical software package R (Version 4.1.2; The R Foundation, Free Software Foundation) for calculating the ICC. We also used Matlab (MathWorks, Natick, MA, USA) for linear regression fitting, correlation coefficient determinations, and paired t-test calculation.

## Results

### ***Pharmacological effects of ***^***11***^***C-PHNO***

Although the administered mass of ^11^C-PHNO (18–24 ng/kg) was below the threshold of adverse effect (29 ng/kg) [22], two out of 8 subjects experienced transient mild nausea within a few minutes following ^11^C-PHNO injection. There was not clear difference between the ^11^C-PHNO mass injected to those subjects who reported nausea (19 $\pm$ 1 ng/kg) and those who did not (21 $\pm$2 ng/kg).

### Imaging results

**Fig. 1:**
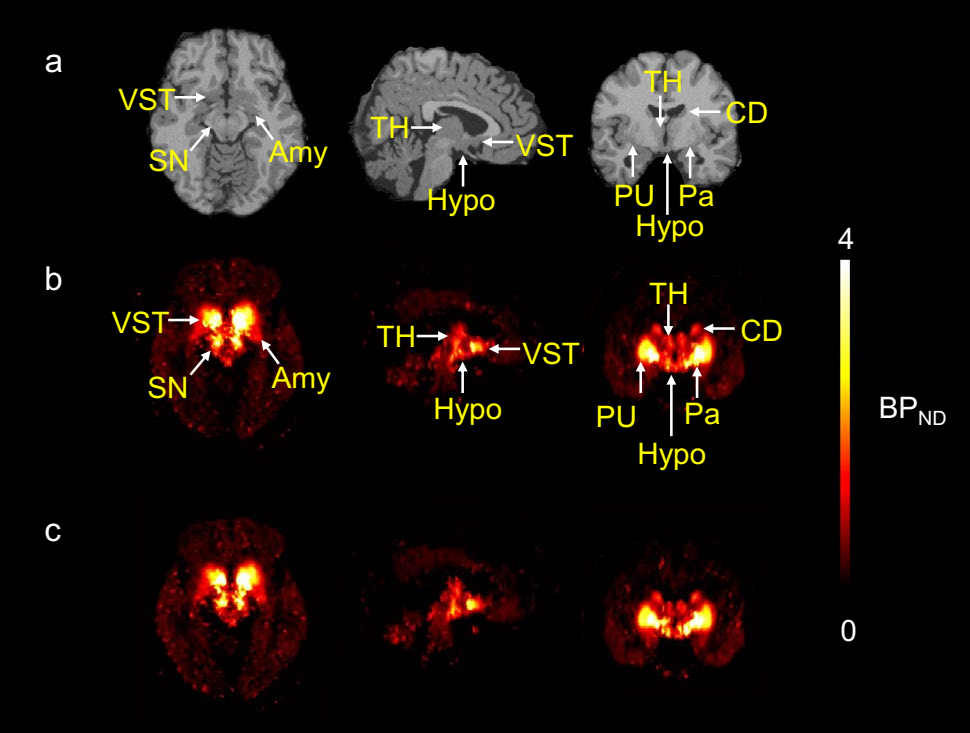
MRI (MNI template) **a**, normalized ^11^C-PHNO BP_ND_ parametric images derived from a full 120-min scan **b** and those derived from a truncated 90-min scan **c** in a representative subject. ^11^C-PHNO binding is seen in the substantia nigra (SN), ventral striatum (VST), pallidum (Pa), amygdala (Amy), hypothalamus (Hypo), putamen (PU), caudate (CD), and thalamus (TH). Parametric images were calculated using a simplified reference tissue model with the cerebellum used as the reference tissue in PXMOD (version 3.8; PMOD Technologies; Zürich, Switzerland). The parametric image obtained using the 90-min scan data **b** was visually equal to that obtained using the 120-min scan data **c**

**Fig. 2:**
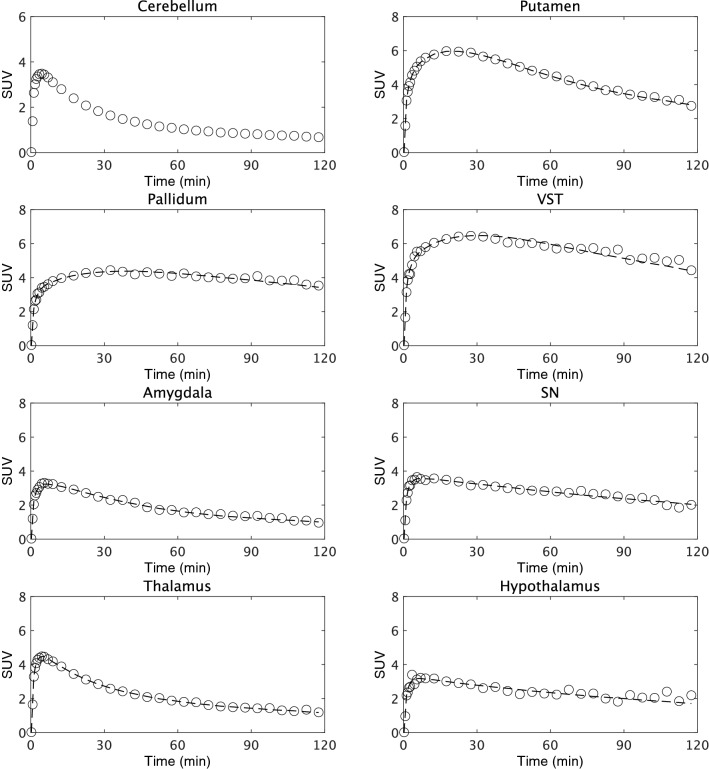
Sample time–radioactivity curve fitting obtained with the simplified reference tissue model (dashed line) in a representative subject’s test scan. Data are derived from standardized uptake values (SUV) in the cerebellum, putamen, pallidum, ventral striatum (VST), amygdala, substantia nigra (SN), thalamus and hypothalamus

Figure 1 shows a typical parametric image of BP_ND_ derived from a full 120-min scan (b) and truncated 90-min scan (c) in a representative subject. The ^11^C-PHNO bindings in the SN, hypothalamus and striatal regions are clearly observed. The parametric image obtained from the 90-min scan was visually equal to that obtained from the 120-min scan. Figure 2 shows the typical TACs and results of curve fitting obtained with the SRTM. Table 2 shows the regional BP_ND_, TRV and ICC obtained from the 120-min scan. There were no significant differences between the test and retest BP_ND_ in any of the ROIs (paired t-test). In the D_3_R-rich regions, the mean relative change of the BP_ND_, namely, the $\mathrm{m}\left(|\Delta {\mathrm{BP}}_{\mathrm{ND}}|\right),$ ranged from 3% (the pallidum and VST) to 9% (the hypothalamus) in the 120-min scan. The ICC values for the BP_ND_ estimates from the 120-min scans exceeded 0.7 in all regions.

**Table 2:** BP_ND_ obtained from the 120-min test–retest scans (*n* = 7 subjects)

Region	Average^1^	$\Delta$ BP_ND_^2^	ICC^3^
Caudate	$1.5 \pm 0.3 (18\mathrm{\%})$	$1\mathrm{\% }\pm 6\mathrm{\% }(5\mathrm{\%})$	0.95 (0.84; 0.99)
Putamen	$2.3 \pm 0.2 (7\mathrm{\%})$	$2\mathrm{\% }\pm 4\mathrm{\% }(3\mathrm{\%})$	0.87 (0.6; 0.96)
Pallidum	$3.2 \pm 0.5 (16\mathrm{\%})$	$-2\mathrm{\% }\pm 4\mathrm{\% }\left(3\mathrm{\%}\right)$	0.96 (0.85; 0.99)
VST	$3.6 \pm 0.3 (8\mathrm{\%})$	$0\mathrm{\% }\pm 4\mathrm{\% }\left(3\mathrm{\%}\right)$	0.85 (0.57; 0.96)
Amygdala	$0.4 \pm 0.1 (18\mathrm{\%})$	$2\mathrm{\% }\pm 4$% (3%)	0.9 (0.69; 0.97)
SN	$1.9 \pm 0.3 (17\mathrm{\%})$	$2\mathrm{\% }\pm 6\mathrm{\% }(5\mathrm{\%})$	0.92 (0.76; 0.98)
Thalamus^4^	$0.6 \pm 0.1 (11\mathrm{\%})$	$-2\mathrm{\% }\pm 10$% (9%)	0.7 (0.13; 0.93)
Hypothalamus	$1.0 \pm 0.3 (25\mathrm{\%})$	$-2\mathrm{\% }\pm 12$% (9%)	0.87 (0.6; 0.96)

^1^Data are presented as mean $\pm$ SD (relative SD) across subjects

^2^Data are presented as m ($\Delta$ BP_ND_) $\pm$ $\upsigma$($\Delta$ BP_ND_) (m|$\Delta$ BP_ND_|)

^3^ICC is presented as an estimate, with the lower and upper bounds of the 95% confidence interval shown in parentheses

^4^Excluding 1 outlier

*VST* ventral striatum, *SN* substantia nigra

### Effects of scan-time shortening

**Fig. 3:**
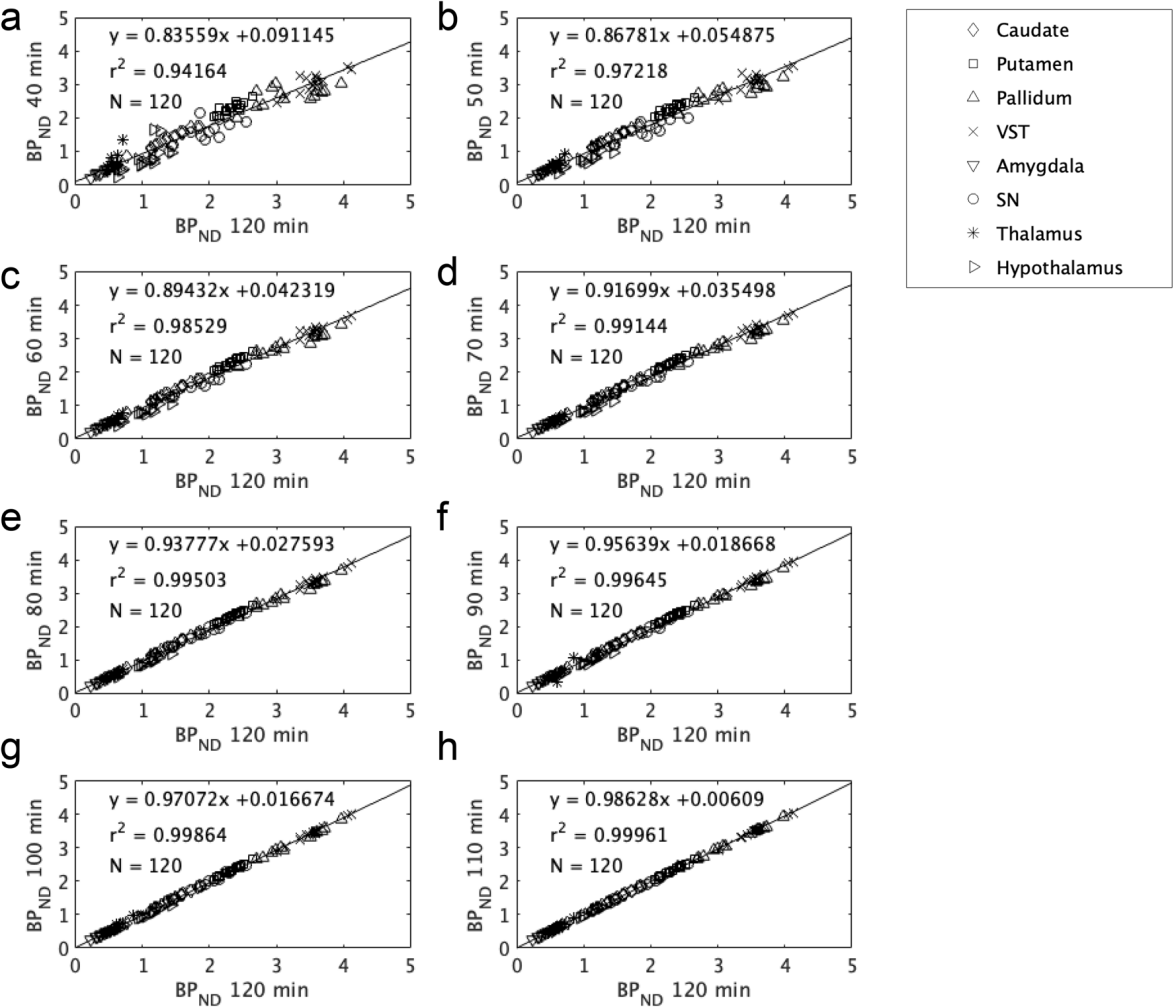
BP_ND_ values obtained with shorter scan times versus those obtained from the 120-min scans (*n* = 15 scans): **a** 40 min; **b** 50 min; **c** 60 min; **d** 70 min; **e** 80 min; **f** 90 min; **g** 100 min; and **h** 110 min. The regression line is shown as a solid line. The number of plotted points (N) was 120. *VST* ventral striatum, *SN* substantia nigra

Figure 3 shows the overall correlations of the BP_ND_ values obtained from the 40- to 110- min scans with those derived from the 120-min scans. Although the coefficient of determination r^2^ deteriorated as the scan time decreased, the r^2^ was as high as 0.94 even in the 40-min scans and 0.996 in the 90-min scans. The regional r^2^, slopes, and intercepts of the regression line for shorter scan times are shown in Supplementary Table 1. In the pallidum, VST, SN and hypothalamus, which are D_3_R-rich regions, the excellent correlation (r^2^ $>$ 0.98) was observed in 90-min scan.

**Fig. 4:**
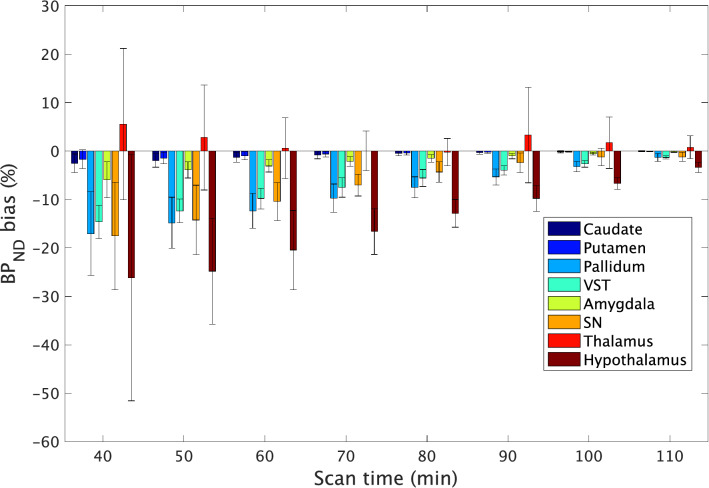
BP_ND_ estimation bias obtained with shorter scan times (*n* = 15 scans). Error bars indicate the SD. *VST* ventral striatum, *SN* substantia nigra

The results of BP_ND_ bias in scans of shorter durations are shown in Fig. 4. Negative BP_ND_ bias increased as the scan time decreased, indicating that the degree of underestimation would increase as the scan time became shorter. In the pallidum, VST and SN, truncation to a 90 min scan time resulted in a bias within 5%. In the hypothalamus, the truncation of scan-time less than 110 min caused a bias larger than 5%.

**Fig. 5:**
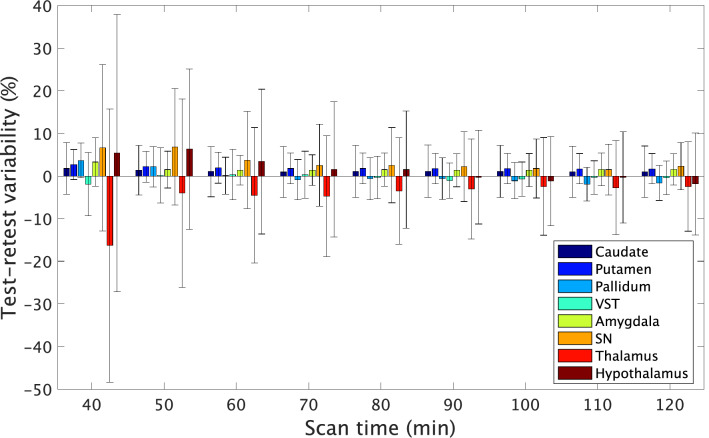
Regional BP_ND_ test–retest variability obtained with shorter scan times (*n* = 7 subjects). Error bars indicate SD. *VST* ventral striatum, *SN* substantia nigra

**Fig. 6:**
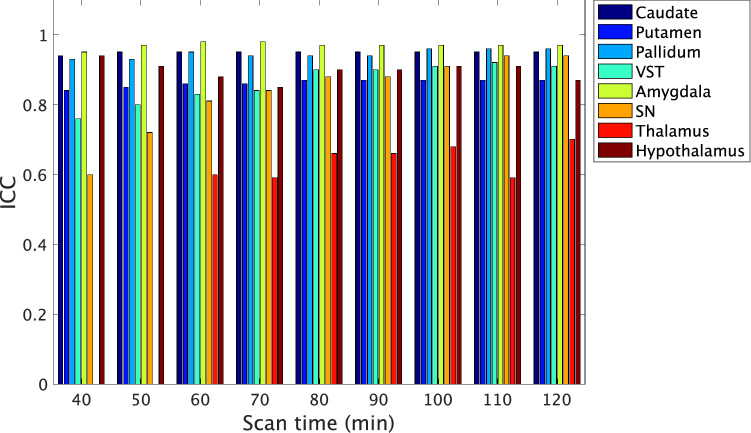
ICC of BP_ND_ obtained with shorter scan times (*n* = 7 subjects). The ICC values in the thalamus obtained from scan of 40- and 50-min durations are not shown, because reliable values were not obtained. *VST* ventral striatum; *SN* substantia nigra

Figures. 5, 6 show the effects of shorter scan times on the TRV and ICC, respectively. Overall, both the TRV and ICC deteriorated as the scan time became shorter. The TRV and ICC values obtained from scans of shorter durations are shown in Supplementary Table 1. The TRV of all regions was smaller than those of the previous report [18], even in 40 min. ICC exceeds 0.7 in 50 min scan in all areas except the thalamus. The minimum scan time for reliable estimate was 40, 50, 90, 90, 50, 80, 120, and 110 min for the caudate, putamen, pallidum, VST, amygdala, SN, thalamus, and hypothalamus, respectively. In the SN, good test–retest reliability of 90-min scans was obtained, with a TRV of 7% and ICC of 0.88. In the pallidum and VST, high test–retest reliability was noted even for 90-min scanning, with a TRV of 3% and ICC of 0.9. Scan times of longer than 90 min did not improve the TRV in these regions, but slightly improved the ICC. In the hypothalamus, a scan duration of more than 90-min scan was needed to maintain high reliability, with a TRV of 9% and ICC of 0.9. In the caudate and putamen, which are D_2_R-rich regions, a TRV of 5% and an ICC of 0.85 were obtained even for a scan duration of 50 min.

### SUVR analysis

The SUVR–1 against BP_ND_ was shown in Supplementary Fig. 1. There was a linear correlation between SUVR–1 and BP_ND_ in the SN, pallidum, and caudate. TRV and ICC were shown in Supplementary Table 2. TRV and ICC of SUVR–1 were comparable to those obtained from BP_ND_.

## Discussion

In the present study, we examined the test–retest reliability of 120-min ^11^C-PHNO PET scans in 7 healthy human volunteers using a whole-body digital PET system, and evaluated the effects of shorter scan times on the estimated BP_ND_ values. Although the test–retest reliability of ^11^C-PHNO has already been studied using a brain-dedicated PET system [18, 20, 21], it has been more than 20 years since these PET devices were introduced and these are no longer commercially available at present. Therefore, re-evaluation of the reliability of ^11^C-PHNO PET measurements using a whole-body PET/CT system, which is the equipment generally available currently around the world, is needed. Because the volumes of D_3_R-rich regions in the brain, e.g., the SN and hypothalamus, are small, BP_ND_ measurement in these regions using a whole-body PET system could be affected by partial volume effect and noise. The special resolution and system sensitivity of the whole-body PET system used in the present study was lower as compared with those of the brain-dedicated PET system used in previous studies. The FWHM and system sensitivity of the Biograph Vision equipment used in the present study were 3.5 mm and 1.6%, whereas those of the brain-dedicated PET system used in the previous test–retest studies were 2.6 mm and 2.5% [23, 33]. The upper mass dose of ^11^C-PHNO is limited by side effects and therefore the administrable dose depends on the specific radioactivity. In the present study, the injected radioactivity dose (137 MBq$\pm 14$) was more than twofold lower than that in previous studies [18, 20]. The lower spatial resolution, lower system sensitivity of the PET system, and lower injected radioactivity could affect the BP_ND_ and TRV estimation; however, in the present study, measurements, including in the SN, obtained using the latest digital whole-body PET/CT system were comparable to those reported from previous studies [18, 20]. Applying the same ROI definition, the obtained BP_ND_ and TRV values derived from 120-min scan in the SN were 1.9 and 5% in the present study, and 2.0 and 20% in one previous study, respectively [18]. These findings suggest superior reliability of the present whole-body PET scanner as compared with the brain-dedicated PET scanner. In the aforementioned previous study, the carry-over mass effect of ^11^C-PHNO could have affected the TRV of BP_ND_, because the test–retest scan interval was 5 h. The administered mass dose of ^11^C-PHNO (25 ng/kg) was close to 50% of the median effective dose of D_3_R, so that the ∆BP_ND_ in the SN was estimated to be 14%. Since the scan interval was greater than 6 days in the present study, we could consider the carry-over effect as being negligible. Furthermore, a diurnal effect of dopamine receptor binding in humans measured by ^11^C-raclopride and ^11^C-FLB-457 PET has been reported [34]. In the present study, ^11^C-PHNO was injected between 2:30 and 3:00 pm in all examinations, whereas in the previous study, the first injections were around 10:00 am and the second injections around 3:00 pm and 4:00 pm. Therefore, the lower test–retest reliability of some regions in the previous study may be attributable to carry-over and diurnal effects, which were negligible in the present study.

Longer scan times pose a challenge for subjects, even for healthy volunteers. Furthermore, also in patients with neurodegenerative or psychiatric diseases, it would be desirable to use shorter scan times for quantitative evaluation of D_3_R binding by PET. The effect of scan-time shortening on BP_ND_ estimation using ^11^C-PHNO PET [17, 18] has not been fully investigated until date. Ginovart et al. used BP_ND_ bias obtained from a single PET scan in 6 normal subjects and compared the striatal BP_ND_ value derived from a truncated scan with that obtained derived from a full 90-min scan [17]. They showed that the BP_ND_ in the pallidum and VST reached stable values at scan durations equal to or greater than 70 and 80 min, respectively. However, they did not evaluate the effect of shortening of the scan time in longer than 90-min scans or analyze D_3_R-rich extrastriatal regions, such as the SN and hypothalamus. We analyzed various truncated data from the full 120-min scan and found that the negative bias of BP_ND_ increased as the scan times decreased in all regions, except the thalamus (Fig. 4). Because the slope of the TAC becomes less steep in the later phase of scanning, truncation in the later phase could cause overestimation of efflux of the tracer, which could result in underestimation of the BP_ND_.

In the present study, we analyzed the effect of shorter scan times on the test–retest reliability, including extrastriatal dopaminergic regions. Our results revealed that a scan duration of 90 min was sufficient to achieve a bias of within 5% and TRV of 7% in the SN, but that a scan duration of 110 min was required to achieve a bias of within 5% and TRV of 9% in the hypothalamus. Gallezot et al. performed a detailed test–retest study of 120-min scans [18]. They compared the BP_ND_ of D_3_R-rich regions, including the SN and hypothalamus, derived from a full 120-min scan with those derived from a 90-min scan, to evaluate the TRV and correlations between the values obtained from the two scan times. Their finding that the BP_ND_ obtained from the 90-min scans was slightly lower than that obtained from the 120-min scans, and that the $\upsigma$($\Delta$ BP_ND_) in the SN was lower in the 120-min scans was consistent with the present results. However, they did not fully analyze the effects of various shorter scan durations. In the present study, we used the determination coefficient, TRV, and ICC for BP_ND_ as measures of the reliability, and estimated the effects of truncation in the later phase (40–120 min) with reference to the full 120-min scan. We evaluated the scan time required for reliable BP_ND_ estimation in the striatal and extrastriatal D_3_R-rich regions. We found that a scan time of 90 min was sufficient to obtain the same values of TRV and ICC in the pallidum, VST and SN as those obtained from 120-min scans in a previous study using brain-dedicated PET systems (3–7% and 0.88–0.94 versus 8–20% and 0.74–0.86, respectively).

^11^C-PHNO binding potentials in the SN and striatum have been used for D_3_R-acting drug occupancy studies [35–38]. There is no definite threshold for the TRV required for drug occupancy studies [39]. Naganawa et al. examined the TRV of the vasopressin receptor radioligand ^11^C-TASP699 and performed a vasopressin receptor antagonist TS-121 occupancy study. They reported that the TRV was within 11% and concluded that this tracer would be a valuable tool for quantifying vasopressin receptor availability [40]. The TRV in the D_3_R-rich striatum and the SN derived from 90-min scans was within 7%, which is considered as being sufficient for D_3_R occupancy studies.

We found a linear correlation between BP_ND_ and SUVR–1 in the SN, pallidum, and caudate. TRV and ICCs of SUVR were comparable to those of BP_ND_. The scan time could potentially be shortened to 30 min to evaluate the bindings of ^11^C-PHNO to dopamine receptors in these regions. However, the previous simulation study of ^18^F-FE-PE2I PET has reported that the relationship between SUVR and BP_ND_ depends on the value of BP_ND_ and the rate constant for the transfer from plasma to the non-displaceable compartment K_1_ [41]. Further evaluation is required in ^11^C-PHNO PET before applying the SUVR method to drug occupancy studies, in which BP_ND_ value has a wide range or the disease shows various K_1_ values among individuals.

The ICCs of BP_ND_ in the thalamus were relatively low compared with other regions due to poor identifiability. The shape of the time activity curve of the thalamus was quite similar to that of the reference region. Therefore, in the SRTM, it was difficult to obtain BP_ND_ and the rate constant for transfer from tissue to plasma compartment (k_2_) independently, which causes poor identifiability of BP_ND_.

In the present study, we did not perform arterial blood sampling to avoid invasive procedures in the volunteers. In a previous study of ^11^C-PHNO PET with a scan duration of 120 min, the BP_ND_ in the striatum and SN derived from the SRTM, with the cerebellum used as the reference region, showed excellent correlation with the BP_ND_ derived from the volume of distribution determined by arterial blood sampling [18]. Therefore, we used BP_ND_ obtained from a 120-min scan as the reference standard for evaluating the effect of scan-time shortening.

## Conclusion

The shortening of ^11^C-PHNO PET scan-time caused slightly decreased correlation with reference BP_ND_ and worsening of test–retest reliability, although in most of D_3_R-rich regions, scan-time truncation to 90-min scan still yielded the test–retest reliability comparable to those derived from 120-min scans using the brain-dedicated PET systems. These findings suggest that the ^11^C-PHNO BP_ND_ of D_3_R-rich regions can be feasibly evaluated from 90-min scans using a digital whole-body PET system.

## Supplementary Information

Below is the link to the electronic supplementary material.

Supplementary material 1 (PDF 174 kb)

Supplementary material 2 (PDF 140 kb)

Supplementary material 3 (PDF 76 kb)

## Data Availability

The data that support the findings of this study are available from the corresponding author upon reasonable request.
