# Supplementary material for: Effect of scan-time shortening on the 11C-PHNO binding potential to dopamine D3 receptor in humans and test–retest reliability
Source: Ann Nucl Med. 2023 Jan 19;37(4):227–37. doi: 10.1007/s12149-022-01819-4 (PMC10060283; doi:10.1007/s12149-022-01819-4)
Supplement: Supplementary file 1 — Supplementary material 1 (PDF 174 kb) [file 12149_2022_1819_MOESM1_ESM.pdf]

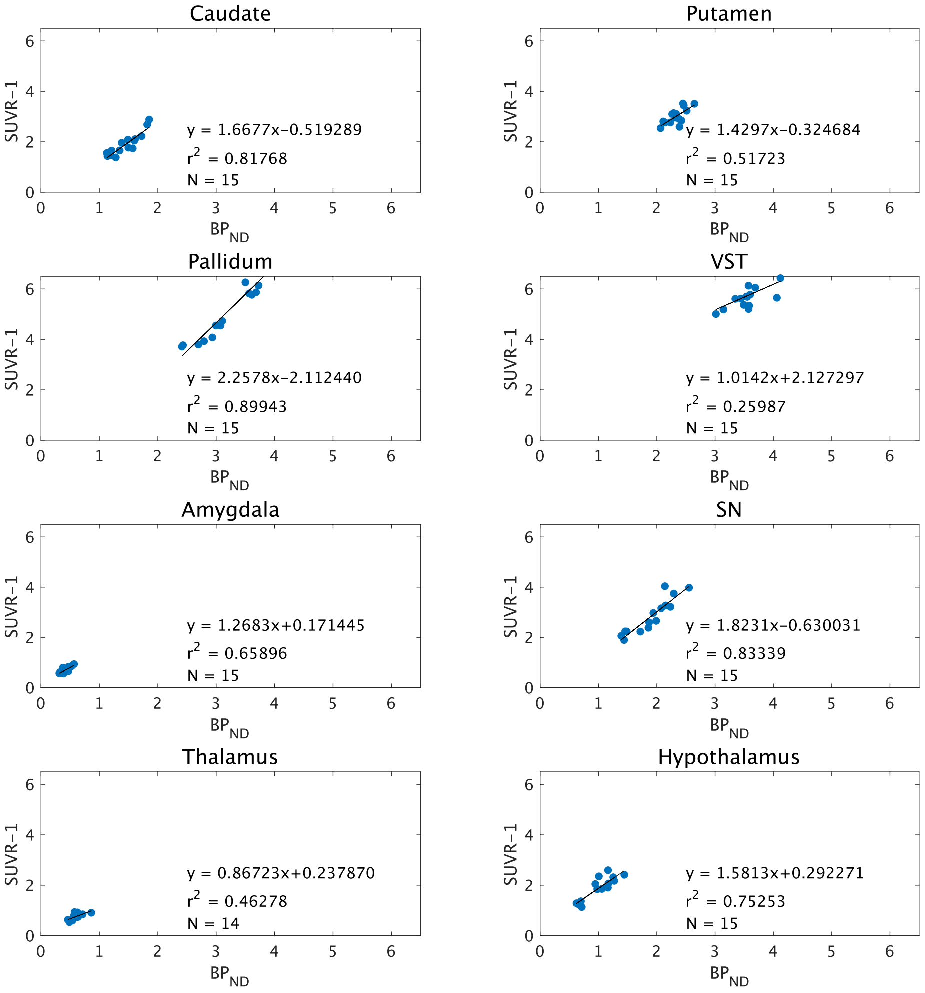

Supplementary Fig. 1: BP_{ND} values versus Standardized uptake value ratio (SUVR) - 1.

VST: ventral striatum; SN: substantia nigra. One outlier was excluded in the analysis of the thalamus.
