# Supplementary material for: Effect of scan-time shortening on the 11C-PHNO binding potential to dopamine D3 receptor in humans and test–retest reliability
Source: Ann Nucl Med. 2023 Jan 19;37(4):227–37. doi: 10.1007/s12149-022-01819-4 (PMC10060283; doi:10.1007/s12149-022-01819-4)
Supplement: Supplementary file 2 — Supplementary material 2 (PDF 140 kb) [file 12149_2022_1819_MOESM2_ESM.pdf]

Title: Effect of scan-time shortening on the ^{11}C-PHNO binding potential to dopamine D_3 receptor in humans and test-retest reliability

Journal name: Annals of Nuclear Medicine

Author names: Keiko Matsunaga[1], Misato Tonomura[1,2], Kohji Abe[1,2] and Eku Shimosegawa[1]

[1] Department of Molecular Imaging in Medicine, Graduate School of Medicine, Osaka University, 2-1, Yamadaoka, Suita, Osaka, 565-0871, Japan

[2] Shionogi & Co., Ltd., 1-8, Doshomachi 3-chome, Chuo-ku, Osaka, 541-0045, Japan

Corresponding author: Keiko Matsunaga

E-mail: matsunaga.keiko.med@osaka-u.ac.jp

Supplementary Table 1: Regional BP_{ND}, TRV, ICC, determination coefficient (r^2), slopes and intercepts of the regression line for shortened scan times (n = 7 subjects)

Scan time	Region	Average[1]	ΔBP_{ND}[2]	ICC[3]	r^2	Slope	Intercept
40 min	Caudate	1.4 ± 0.2 (16%)	$2\% \pm 6\%$ (5%)	0.94 (0.77; 0.98)	0.983	0.877	0.143
	Putamen	2.3 ± 0.2 (7%)	$3\% \pm 4\%$ (4%)	0.84 (0.5; 0.96)	0.795	0.898	0.21
	Pallidum	2.7 ± 0.3 (13%)	$4\% \pm 4\%$ (4%)	0.93 (0.76; 0.98)	0.464	0.428	1.305
	VST	3.0 ± 0.3 (9%)	$-2\% \pm 7\%$ (6%)	0.76 (0.31; 0.94)	0.7	0.857	-0.001
	Amygdala	0.4 ± 0.1 (20%)	$3\% \pm 6\%$ (5%)	0.95 (0.83; 0.99)	0.973	1.051	-0.047
	SN	1.6 ± 0.3 (20%)	$7\% \pm 19\%$ (17%)	0.6 (0.02; 0.89)	0.637	0.789	0.058

	Thalamus[4]	0.6 ± 0.0 (6%)	−16% ± 32% (26%)	-0.14 (-0.74; 0.65)	0.443	2.324	-0.68
	Hypothalamus	0.8 ± 0.4 (55%)	5% ± 32% (22%)	0.94 (0.79; 0.99)	0.498	1.124	-0.38
50 min	Caudate	1.4 ± 0.2 (17%)	1% ± 6% (5%)	0.95 (0.81; 0.99)	0.994	0.928	0.075
	Putamen	2.3 ± 0.2 (7%)	2% ± 4% (3%)	0.85 (0.53; 0.96)	0.942	0.952	0.082
	Pallidum	2.7 ± 0.3 (13%)	2% ± 5% (4%)	0.93 (0.75; 0.98)	0.785	0.572	0.914
	VST	3.1 ± 0.3 (8%)	0% ± 6% (5%)	0.8 (0.39; 0.95)	0.735	0.777	0.365
	Amygdala	0.4 ± 0.1 (19%)	1% ± 4% (4%)	0.97 (0.9; 0.99)	0.995	1.046	-0.036
	SN	1.6 ± 0.3 (19%)	7% ± 14% (14%)	0.72 (0.23; 0.93)	0.821	0.884	-0.055
	Thalamus[4]	0.6 ± 0.1 (18%)	−4% ± 22% (18%)	-0.09 (-0.72; 0.68)	0.669	1.424	-0.23
	Hypothalamus	0.8 ± 0.3 (34%)	6% ± 19% (15%)	0.91 (0.68; 0.98)	0.724	0.849	-0.095
60 min	Caudate	1.4 ± 0.2 (17%)	1% ± 6% (5%)	0.95 (0.82; 0.99)	0.997	0.954	0.047

	Putamen	2.3 ± 0.2 (7%)	2% ± 4% (3%)	0.86 (0.54; 0.96)	0.978	0.978	0.031
	Pallidum	2.8 ± 0.4 (14%)	0% ± 4% (3%)	0.95 (0.83; 0.99)	0.939	0.701	0.575
	VST	3.2 ± 0.3 (8%)	0% ± 6% (5%)	0.83 (0.47; 0.96)	0.876	0.859	0.154
	Amygdala	0.4 ± 0.1 (19%)	1% ± 3% (3%)	0.98 (0.92; 1)	0.997	1.023	-0.023
	SN	1.7 ± 0.3 (19%)	4% ± 11% (10%)	0.81 (0.41; 0.95)	0.94	0.953	-0.11
	Thalamus[4]	0.6 ± 0.1 (15%)	−5% ± 16% (14%)	0.6 (-0.05; 0.9)	0.826	1.206	-0.116
	Hypothalamus	0.8 ± 0.2 (30%)	3% ± 17% (13%)	0.88 (0.59; 0.97)	0.808	0.836	-0.04
70 min	Caudate	1.4 ± 0.2 (17%)	1% ± 6% (5%)	0.95 (0.82; 0.99)	0.998	0.973	0.027
	Putamen	2.3 ± 0.2 (7%)	2% ± 4% (3%)	0.86 (0.56; 0.97)	0.99	0.996	-0.002
	Pallidum	2.9 ± 0.4 (14%)	−1% ± 5% (4%)	0.94 (0.8; 0.99)	0.966	0.756	0.48
	VST	3.3 ± 0.3 (8%)	0% ± 6% (5%)	0.84 (0.49; 0.96)	0.914	0.866	0.207

	Amygdala	0.4 ± 0.1 (19%)	$1\% \pm 4\%$ (3%)	0.98 (0.91; 0.99)	0.998	1.018	-0.017
	SN	1.7 ± 0.3 (17%)	$3\% \pm 10\%$ (8%)	0.84 (0.5; 0.96)	0.973	0.949	-0.04
	Thalamus[4]	0.6 ± 0.1 (13%)	$-5\% \pm 14\%$ (12%)	0.59 (-0.06; 0.9)	0.9	1.109	-0.063
	Hypothalamus	0.8 ± 0.2 (26%)	$2\% \pm 16\%$ (12%)	0.85 (0.52; 0.96)	0.925	0.835	-0.001
80 min	Caudate	1.4 ± 0.2 (17%)	$1\% \pm 6\%$ (5%)	0.95 (0.81; 0.99)	0.999	0.982	0.018
	Putamen	2.3 ± 0.2 (7%)	$2\% \pm 4\%$ (3%)	0.87 (0.56; 0.97)	0.996	1.001	-0.01
	Pallidum	3.0 ± 0.4 (14%)	$-1\% \pm 5\%$ (3%)	0.94 (0.8; 0.99)	0.979	0.811	0.374
	VST	3.3 ± 0.3 (9%)	$0\% \pm 5\%$ (4%)	0.9 (0.67; 0.98)	0.956	0.951	-0.027
	Amygdala	0.4 ± 0.1 (18%)	$2\% \pm 4\%$ (3%)	0.97 (0.9; 0.99)	0.999	1.014	-0.012
	SN	1.8 ± 0.3 (17%)	$3\% \pm 9\%$ (8%)	0.88 (0.6; 0.97)	0.981	0.951	0.007
	Thalamus[4]	0.6 ± 0.1 (13%)	$-4\% \pm 12\%$ (11%)	0.66 (0.06; 0.92)	0.95	1.072	-0.043

	Hypothalamus	0.9 ± 0.2 (25%)	1% ± 14% (11%)	0.9 (0.67; 0.98)	0.967	0.834	0.036
90 min	Caudate	1.4 ± 0.3 (17%)	1% ± 6% (5%)	0.95 (0.81; 0.99)	1	0.988	0.012
	Putamen	2.3 ± 0.2 (7%)	2% ± 4% (3%)	0.87 (0.56; 0.97)	0.998	1	-0.005
	Pallidum	3.0 ± 0.5 (15%)	−1% ± 5% (3%)	0.94 (0.8; 0.99)	0.991	0.86	0.283
	VST	3.4 ± 0.3 (9%)	−1% ± 4% (3%)	0.9 (0.67; 0.98)	0.982	0.964	-0.016
	Amygdala	0.4 ± 0.1 (18%)	1% ± 4% (3%)	0.97 (0.9; 0.99)	0.999	1.01	-0.009
	SN	1.8 ± 0.3 (16%)	2% ± 8% (7%)	0.88 (0.6; 0.97)	0.985	0.953	0.038
	Thalamus[5]	0.6 ± 0.1 (12%)	−3% ± 12% (10%)	0.66 (0.06; 0.92)	0.739	1.461	-0.284
	Hypothalamus	0.9 ± 0.2 (25%)	0% ± 11% (9%)	0.9 (0.67; 0.98)	0.975	0.859	0.042
100 min	Caudate	1.5 ± 0.3 (17%)	1% ± 6% (5%)	0.95 (0.82; 0.99)	1	0.994	0.006
	Putamen	2.3 ± 0.2 (7%)	2% ± 4% (3%)	0.87 (0.57; 0.97)	0.999	0.998	0.002

	Pallidum	3.1 ± 0.5 (16%)	−1% ± 4% (3%)	0.96 (0.84; 0.99)	0.997	0.917	0.163
	VST	3.5 ± 0.3 (9%)	−1% ± 4% (3%)	0.91 (0.7; 0.98)	0.993	0.99	-0.059
	Amygdala	0.4 ± 0.1 (18%)	1% ± 4% (3%)	0.97 (0.89; 0.99)	1	1.005	-0.005
	SN	1.8 ± 0.3 (17%)	2% ± 7% (6%)	0.91 (0.71; 0.98)	0.99	0.961	0.047
	Thalamus[5]	0.6 ± 0.1 (12%)	−2% ± 11% (10%)	0.68 (0.1; 0.93)	0.951	1.266	-0.148
	Hypothalamus	1.0 ± 0.2 (25%)	−1% ± 10% (9%)	0.91 (0.7; 0.98)	0.99	0.896	0.035
110 min	Caudate	1.5 ± 0.3 (17%)	1% ± 6% (5%)	0.95 (0.82; 0.99)	1	0.997	0.003
	Putamen	2.3 ± 0.2 (7%)	2% ± 4% (3%)	0.87 (0.57; 0.97)	1	0.999	0.002
	Pallidum	3.1 ± 0.5 (16%)	−2% ± 4% (3%)	0.96 (0.84; 0.99)	0.998	0.95	0.115
	VST	3.5 ± 0.3 (9%)	0% ± 4% (3%)	0.92 (0.73; 0.98)	0.998	1.005	-0.063
	Amygdala	0.4 ± 0.1 (18%)	2% ± 4% (3%)	0.97 (0.89; 0.99)	1	1.003	-0.002

SN	1.8 ± 0.3 (17%)	1% ± 6% (5%)	0.94 (0.79; 0.99)	0.997	0.987	0.002
Thalamus[5]	0.6 ± 0.1 (12%)	−3% ± 11% (9%)	0.59 (-0.15; 0.92)	0.987	1.118	-0.066
Hypothalamus	1.0 ± 0.2 (25%)	0% ± 11% (9%)	0.91 (0.69; 0.98)	0.993	0.933	0.031

[1]Data are presented as mean ± SD (relative SD) across subjects.

[2]Data are presented as m (ΔBP_{ND}) ± $\sigma(\Delta BP_{ND})$ ($m|\Delta BP_{ND}|$).

[3]ICC is presented as an estimate, with the lower and upper bounds of the 95% confidence interval shown in parentheses.

[4]Exclude 4 outliers. n = 3 subjects.

[5]Exclude 1 outlier. n = 6 subjects.

VST: ventral striatum; SN: substantia nigra.
