# Supplementary material for: Effect of scan-time shortening on the 11C-PHNO binding potential to dopamine D3 receptor in humans and test–retest reliability
Source: Ann Nucl Med. 2023 Jan 19;37(4):227–37. doi: 10.1007/s12149-022-01819-4 (PMC10060283; doi:10.1007/s12149-022-01819-4)
Supplement: Supplementary file 3 — Supplementary material 3 (PDF 76 kb) [file 12149_2022_1819_MOESM3_ESM.pdf]

Supplementary Table 2: Standardized uptake value ratio (SUVR) -1 over 90-120 min obtained from test-retest scans (n = 7 subjects)

Region	Average[1]	ΔBP_{ND}[2]	ICC[3]
Caudate	1.9 ± 0.4 (23%)	0.7% ± 2.8% (3%)	0.90 (0.65; 0.97)
Putamen	3.0 ± 0.3 (10%)	1.2% ± 1.7% (2%)	0.73 (0.25; 0.93)
Pallidum	5.0 ± 1.1 (22%)	−0.1% ± 1.2% (1%)	0.98 (0.93; 1)
VST	5.7 ± 0.6 (10%)	1.1% ± 1.9% (2%)	0.57 (-0.02; 0.88)
Amygdala	0.7 ± 0.1 (16%)	1.9% ± 3.0% (2%)	0.64 (0.09; 0.90)
SN	2.8 ± 0.6 (23%)	1% ± 0.9% (1%)	0.97 (0.9; 0.99)
Thalamus	0.7 ± 0.1 (17%)	0.8% ± 1.9% (2%)	0.9 (0.62; 0.98)
Hypothalamus	1.9 ± 0.5 (24%)	0.5% ± 3.2% (3%)	0.87 (0.57; 0.97)

[1]Data are presented as mean ± SD (relative SD) across subjects.

[2]Data are presented as m (Δ(SUVR - 1)) ± σ(Δ(SUVR - 1)) (m|Δ(SUVR - 1)|).

[3]ICC is presented as an estimate, with the lower and upper bounds of the 95% confidence interval shown in parentheses.

VST: ventral striatum; SN: substantia nigra.
